# Modeling of age-related neurological disease: utility of zebrafish

**DOI:** 10.3389/fnagi.2024.1399098

**Published:** 2024-05-03

**Authors:** Tohgo Kanoh, Takamasa Mizoguchi, Ayako Tonoki, Motoyuki Itoh

**Affiliations:** ^1^Graduate School of Pharmaceutical Sciences, Chiba University, Chiba, Japan; ^2^Research Institute of Disaster Medicine, Chiba University, Chiba, Japan; ^3^Health and Disease Omics Center, Chiba University, Chiba, Japan

**Keywords:** memory, aging, mouse, zebrafish, lifespan, cerebral blood vessel, Alzheimer’s disease

## Abstract

Many age-related neurological diseases still lack effective treatments, making their understanding a critical and urgent issue in the globally aging society. To overcome this challenge, an animal model that accurately mimics these diseases is essential. To date, many mouse models have been developed to induce age-related neurological diseases through genetic manipulation or drug administration. These models help in understanding disease mechanisms and finding potential therapeutic targets. However, some age-related neurological diseases cannot be fully replicated in human pathology due to the different aspects between humans and mice. Although zebrafish has recently come into focus as a promising model for studying aging, there are few genetic zebrafish models of the age-related neurological disease. This review compares the aging phenotypes of humans, mice, and zebrafish, and provides an overview of age-related neurological diseases that can be mimicked in mouse models and those that cannot. We presented the possibility that reproducing human cerebral small vessel diseases during aging might be difficult in mice, and zebrafish has potential to be another animal model of such diseases due to their similarity of aging phenotype to humans.

## 1 Introduction

The quest to understand the intricacies of human age-related neurological diseases has led scientists to explore various animal models, each offering unique insights into the pathophysiology of diseases. Among these, the mouse (*Mus musculus*) models have been used as powerful tools in studying neurological disorder.

The mouse model has been a cornerstone in biomedical research for decades. Mice share about 85% of their DNA with humans and have similar nervous systems, making them excellent models for studying the age-related neurological disease (Waterston et al., 2002; Xu et al., 2022). The availability of sophisticated genetic manipulation techniques in mice further enhances their utility in disease modeling. Mouse models of age-related neurological diseases have provided deep insight into these diseases in humans. However, no single model can perfectly recapitulate all aspects of human age-related neurological disease, and it is necessary to combine insights from several models to understand the pathophysiology of these diseases.

Zebrafish (*Danio rerio*), a small tropical freshwater fish, has gained prominence in the scientific community due to its genetic and physiological similarities to humans. Approximately 70% of human genes have at least one obvious zebrafish orthologue, making it a valuable model for studying human diseases (Choi et al., 2021). Moreover, their prolific breeding capabilities allow for the generation of large sample sizes. The transparency of zebrafish larvae or adult zebrafish of mutants lacking melanocytes and iridophores permits live imaging of cellular and molecular processes *in vivo*, providing a dynamic view of disease progression and therapeutic effects that is not easily achievable in other model organisms (White et al., 2008). In addition to these merits, recent studies revealed the similarities of neurological disease-related aging phenotypes between zebrafish and humans, suggesting that zebrafish has a potential as a age-related neurological disease model (Arslan-Ergul et al., 2013).

In this review, we compared the neurological disease-related aging phenotypes that are common or different among humans, mice, and zebrafish. Subsequently, we discuss the advantages and limitations of mouse models for age-related neurological diseases and explore the potential of zebrafish to overcome these limitations.

## 2 The age-associated phenotype between humans, mice, and zebrafish

When considering the creation of model organisms for neurodegenerative diseases accelerated by aging, it is critical to assess whether humans and the model organisms follow similar aging processes, as this can significantly impact the applicability of the research findings to humans. Therefore, it is useful to summarize the similarities and differences in aging phenotypes. Here, we compared the normal aging phenotypes across humans, mice, and zebrafish (Table 1). In mice and zebrafish, aging impairs various physiological functions similar to those in humans, such as basal metabolism (Kitazoe et al., 2019; Yang et al., 2019; Li et al., 2022), locomotor activity (Hunter et al., 2016; Yanai and Endo, 2021; Rutkove et al., 2023), cognitive function (Aartsen et al., 2002; Ruhl et al., 2015; Yang et al., 2018; Yanai and Endo, 2021), bone metabolism (Szulc et al., 2001; Monma et al., 2019; Wan et al., 2021), regenerative capacity (Tsai et al., 2007; Loforese et al., 2017; Rando and Jones, 2021; Tower et al., 2022), and reproductive capacity (Franks and Payne, 1970; Little, 1997; Tsai et al., 2007; Aitken, 2023), indicating a degree of conserved aging mechanisms across these species (Table 1). In leveraging the advantages of each model organism to mimic or overcome human age-related neurological diseases, it is essential to understand differences that may lead to divergent phenotypes with brain aging. The differences in normal age-related phenotypes between mice and zebrafish include a lifespan and clearance systems in the brain.

**Table 1:** The characteristics associated with aging among humans, mice, and zebrafish.

Phenotype	Human	Mouse	Zebrafish	References
Basal metabolism	↓	↓	↓	Yang et al. (2019), Li et al. (2022), and Kitazoe et al. (2019)
Locomotor activity	↓	↓	↓	Rutkove et al. (2023), Yanai and Endo (2021), and Hunter et al. (2016)
Cognitive function	↓	↓	↓	Yang et al. (2018), Yanai and Endo (2021), and Aartsen et al. (2002)
Bone metabolism	↓	↓	↓	Monma et al. (2019), Wan et al. (2021), and Szulc et al. (2001)
Regenerative ability	↓	↓	↓	Tsai et al. (2007), Loforese et al. (2017), and Rando and Jones (2021)
Reproductive capacity	↓	↓	↓	Tsai et al. (2007), Franks and Payne (1970), Little (1997), and Aitken (2023)
Lifespan	70 years (long among similar body mass species)	25 month (short among similar body mass species)	42 month (long among similar body mass species)	Kishi (2004), Roser et al. (2013), and Fahlström et al. (2011)
Aβ clearance	+	+++	+	Mawuenyega et al. (2010), Cirrito et al. (2003), and Jeong et al. (2021)
Heart rate (bpm)	60–70	500	110–130	Wang et al. (2017) and Benveniste et al. (2018)
Cerebral blood flow	↓	→	↓	Mizoguchi et al. (2023), Wei et al. (2020), and Pantano et al. (1984)

Arrows indicate the direction of aging-dependent change (↓: decrease, →: no change). “+” indicates the ability of Aβ clearance; mice have much more efficient capacity of Aβ clearance compared with human and zebrafish.

### 2.1 Lifespan

Zebrafish is known to have an average lifespan of 42 months (Gerhard et al., 2002; Kishi, 2004), while the lifespan of mice typically ranges from 25 to 26 months (Fahlström et al., 2011) (Table 1). Interestingly, even genetically modified mice designed for longevity live as long as the wild-type zebrafish (Bartke et al., 2001). It is known that there is a positive correlation between body weight and a lifespan in vertebrates (Kuparinen et al., 2023), suggesting that zebrafish, despite their relatively small size, is inherently long-lived species compared to other animals including mice. In addition, humans tend to live longer among mammals of equivalent body weight, while mice demonstrate a relatively shorter lifespan (Roser et al., 2013; Kowalczyk et al., 2020) (Table 1). These observations suggest that zebrafish offers a unique opportunity to replicate aging-related phenotypes that are not observable in shorter-lived species. This aspect of zebrafish biology underscores the potential of using them to explore complex aging processes and their implications for human health, offering insights into longevity and mechanisms underlying age-related diseases.

### 2.2 Clearance systems in the brain

In the context of age-related neurological diseases, the efficiency of clearance systems in the brain plays a pivotal role in maintaining brain homeostasis. As organisms age, the efficiency of autophagy and the ubiquitin-proteasome system declines, leading to a disruption in proteostasis and subsequent accumulation of abnormal proteins in the brain (Kaushik and Cuervo, 2015). To clear these substances, the brain has several clearance mechanisms including the glymphatic system, which can drain these substances along cerebral small vessels (Nedergaard, 2013). Benveniste et al. (2018) suggest significant differences in the clearance efficiency of the glymphatic system between humans and mice (Table 1). This assertion is supported by the several studies investigating the half-life of amyloid-beta (Aβ), mainly cleared by the glymphatic system (Wang et al., 2023). In humans with Alzheimer’s disease, the half-life of Aβ is around 13 h, whereas in Alzheimer’s disease model mice, even aged individuals show a half-life of merely 2–4 h, indicating that the clearance of Aβ from the brain in mice is at least three times faster than that in humans (Cirrito et al., 2003; Barten et al., 2005; Mawuenyega et al., 2010; Qosa et al., 2014). Research utilizing zebrafish larvae, in which Aβ was injected into the brain, and the amount of Aβ was measured between 5 h-post-injection and 24 h-post-injection, showed only a 40% reduction in Aβ levels, indicating the half-life of Aβ is over 19 h in zebrafish larvae (Jeong et al., 2021). These studies suggest that zebrafish possesses a capability for brain clearance that is similar to humans, while mice exhibit a significantly higher capacity compared to the other two species (Table 1).

One of the possible causes of this difference is variations in heart rate, which directly impact the efficiency of the glymphatic system. It has been demonstrated that the glymphatic system is stimulated upon artificially elevating the heart rate in mice through the administration of dobutamine (Iliff et al., 2013). The resting heart rate of mice is around 500 bpm, while it is around 60–70 bpm in humans (Wang et al., 2017; Benveniste et al., 2018) (Table 1). Therefore, the clearance capacity in the brain in humans might be much weaker than in mice. Notably, the resting heart rate of adult zebrafish is around 120 bpm (Table 1), suggesting that zebrafish may possess brain clearance mechanisms closer to humans than to mice (Mousavi and Patil, 2020). Moreover, it is implied that dysregulation of cerebral blood flow affects glymphatic system (Sepehrinezhad et al., 2023). The cerebral blood flow is decreased with aging in humans and zebrafish, whereas this does not change in mice (Pantano et al., 1984; Wei et al., 2020; Mizoguchi et al., 2023) (Table 1). This suggests that the efficiency of glymphatic system is decreased with aging in humans and zebrafish, but less in mice.

These could imply that neurovascular aging related to neurodegenerative diseases may occur earlier in humans and zebrafish compared to mice. Thus, considering the difference in brain clearance capacity points toward the potential of zebrafish as a more representative model for studying human cerebrovascular aging and clearance mechanisms in the context of aging-related neurodegenerative diseases.

## 3 The diseases in which human pathology is partially mimicked by mouse models

For some age-accelerated disorders, such as Alzheimer’s disease and cerebral small vessel diseases, mouse models do not fully mimic the human disease. These diseases are known to be related to cerebrovascular pathology, and aging might be a large risk factor of onset and progression of these diseases (described in detail in section 4).

### 3.1 Alzheimer’s disease

Alzheimer’s disease (AD) is the most common type of dementia. Two main pathological hallmarks of AD are Aβ plaques and neurofibrillary tangles (NFT) (Blennow et al., 2006). The Aβ plaques are formed by deposition of Aβ protein, and the NFT is formed by intracellular tau protein hyperphosphorylation, which is induced by Aβ (Blennow et al., 2006). There are several AD risk genes such as *APP*, *PSEN1*, and *PSEN2*. The single missense mutation of these genes causes the AD pathology in human (Bagyinszky et al., 2016).

Transgenic mouse models have significantly contributed to our understanding of Alzheimer’s disease (AD), with multiple types of transgenic mice of AD overexpressing mutant forms of AD risk genes (Sanchez-Varo et al., 2022; Yokoyama et al., 2022). These models exhibit key features of AD pathology, including Aβ plaques and cognitive decline. However, they have limitations, including artificial temporal or spatial expression patterns in transgenic overexpression systems, leading to complex outcomes that may not accurately represent the human condition. For example, as reported by Jankowsky et al. (2005), analyzing cognitive behavior was difficult due to their severe hyperactivity which is not a human AD symptom, using transgenic mice expressing chimeric mouse/human APP Swedish/Indiana (carrying KM570, 571NL, and V617F mutation). The authors described that hyperactivity, which is not observed in human, might be caused by neuronal alterations due to transgene expression during early postnatal development (Jankowsky et al., 2005). Moreover, most transgenic mice, such as single transgenic mice (carrying mutant APP) or double transgenic mice (carrying both mutant APP and mutant PSEN1), did not show the formation of NFT, despite exhibiting cognitive decline (Metaxas and Kempf, 2016; Drummond and Wisniewski, 2017; Sasaguri et al., 2017). These limitations highlight the need for alternative AD models, which substitute for transgenic models.

To overcome the issues associated with transgenic models, researchers have attempted to create models using knock-in techniques. These techniques aim to mimic wild-type expression levels and patterns more closely. However, the expected Aβ plaque deposition was not detected in APP Swedish (carrying KM670, 671NL mutation) or London (carrying V717I mutation), and memory impairment was not observed in the knock-in model that carries a single APP mutation (carrying V642I) (Kawasumi et al., 2004; Köhler et al., 2005). Subsequent efforts led to the development of *APP* knock-in mice models incorporating multiple familial Alzheimer’s mutations. These models include the Swedish (NL), Beyreuther/Iberian (F), and Arctic (G) mutations. The APP NL-G-F mice, which harbor all three mutations, began to develop Aβ plaques at two months and showed memory impairment from six months (Saito et al., 2014). However, further research revealed that in some cases, APP NL-G-F mice did not exhibit the expected decline in memory abilities [refer to the discussion in Sakakibara et al. (2019)]. These suggest that introducing mutated forms of APP through knock-in techniques may not fully replicate the symptoms of AD. In terms of PSEN1/2, there are a few studies on familial mutant PSEN1 knock-in mouse models. These mouse models carry a single familial mutation such as L435F, I213T or R278I, and have shown Aβ plaques but exhibit no or mild memory impairment (Lalonde and Strazielle, 2005; Saito et al., 2011; Xia et al., 2015). Therefore, it is difficult to conclude the PSEN1 knock-in AD mouse models fully replicate the AD pathology.

### 3.2 Cerebral small vessel diseases

Cerebral Small Vessel Diseases (CSVDs) are the collective term for diseases that affect the cerebral small vessels. Damage to small vessels lead to lesions in subcortical structures like lacunar infarcts, white matter lesions, large hemorrhages, and microbleeds, leading to dementia (Pantoni, 2010). The progression of CSVDs is highly age-associated (Chung et al., 2023). Characteristic pathologies of CSVDs include enlarged perivascular spaces and formation of abluminal protein deposits (Pantoni, 2010). Recent studies have suggested that the glymphatic system plays a pivotal role in the pathophysiology of CSVDs (Mestre et al., 2017; Benveniste and Nedergaard, 2022). The mouse models of monogenic CSVDs such as CADASIL, CARASIL, Fabry disease, and RVCL, are discussed in the following section.

Cerebral autosomal dominant arteriopathy with subcortical infarct and leukoencephalopathy (CADASIL) is a prototypical CSVD caused by mutations in the *NOTCH3* gene (Chabriat et al., 2009; André, 2010). CADASIL is characterized by the accumulation of granular osmiophilic material (GOM) and the extracellular domain of NOTCH3 in the vascular walls, leading to the loss of perivascular cell (vascular smooth muscle cell and pericyte), vascular dysfunction, recurrent lacunar infarcts, cognitive impairments, depressive symptoms, and motor deficits (Kalaria et al., 2004; Chabriat et al., 2009; Dziewulska and Lewandowska, 2012). Despite its clinical importance, the precise pathogenesis of CADASIL remains elusive, and there is currently no effective treatment, emphasizing the need for animal models to better understand and address this condition.

Several transgenic mouse models have been developed that can partially mimic CADASIL pathology. Transgenic mice with the rat Notch3 R169C mutation exhibit GOM lesions, Notch3 accumulation, pericyte loss, and memory impairment (Joutel et al., 2010; Ghosh et al., 2015; Ehret et al., 2021). Mice with the human NOTCH3 R90C mutation also showed GOM lesions, vascular dysfunction, and memory deficits (Ruchoux et al., 2003; Lacombe et al., 2005; Liu et al., 2015). However, Ruchoux et al. (2003) showed that these mice did not exhibit significant brain parenchyma damage. These suggest that the observed memory deficits might arise from mechanisms different from those in human CADASIL. Another transgenic model expressing the human NOTCH3 R182C mutation has been established, which develops GOM lesions, but does not show the white matter lesions, changes in cerebral blood flow, or memory impairment seen in human patients (Rutten et al., 2015; Gravesteijn et al., 2020). To more accurately mimic the pathological conditions of human CADASIL, knock-in models are increasingly developed. There are two types of knock-in CADASIL mouse models; one is Notch3 R170C (corresponding to human R169C) knock-in mice, and another is Notch3 R142C (corresponding to human R141C) knock-in mice (Lundkvist et al., 2005; Wallays et al., 2011). However, it is important to note that, to our knowledge, none of these CADASIL knock-in mouse models have yet exhibited memory impairments.

Cerebral autosomal recessive arteriopathy with subcortical infarcts and leukoencephalopathy (CARASIL) is a hereditary disease caused by loss-of-function mutations in the *Htra1* gene, characterized by baldness, strokes, white matter lesions, and early-onset dementia (Tikka et al., 2014). The abnormal accumulation of extracellular matrix proteins and TGF-β1, which are degraded by Htra1 was observed around small cerebral arteries in CARASIL patients (Hara et al., 2009). While aged *Htra1* knockout mice showed the abnormal protein accumulation in cerebral arteries, they have not successfully replicated white matter lesions, strokes, or smooth muscle cell loss seen in the human condition (Beaufort et al., 2014; Kato et al., 2021).

Fabry disease results from mutations in the gene encoding α-galactosidase A (α-GalA), a lysosomal hydrolase enzyme (Germain, 2010). This leads to decreased enzyme activity and the accumulation of its substrate, globotriaosylceramide (GL-3), within the lysosomes of various organs, including blood vessels, kidneys, heart, and dorsal root ganglia (Choi, 2015). The primary symptoms of Fabry disease in humans are burning pain, autonomic dysfunctions, posterior circulation stroke, cognitive impairment, and depression (Bolsover et al., 2014; Choi, 2015). α-GalA knockout mice have been developed to study Fabry disease. While these mice exhibit the accumulation of GL-3 (Ohshima et al., 1997; Bangari et al., 2015), they did not show depressive-like behavior or learning and memory deficits (Hofmann et al., 2017).

Retinal vasculopathy with cerebral leukodystrophy (RVCL) is caused by mutations in a 3′-5′ DNA exonuclease TREX1 (Richards et al., 2007). The primary symptoms in human RVCL patients include activation of immune system, leukoencephalopathy, lacunar infarcts, retinopathy, nephropathy, and migraines (Schuh et al., 2015; Søndergaard et al., 2017). While the pathomechanism remains unknown, vascular basement membranes were found to be thicker and multi-layered (Søndergaard et al., 2017). This suggests that the clearance system in the brain might be impaired in RVCL patients. Frame-shift mutant TREX1 knock-in mice have been developed as RVCL models. Although these mice replicated activation of immune system, they did not exhibit key manifestations such as retinopathy and neurological symptoms (Sakai et al., 2017).

## 4 The diseases in which human pathology is mimicked by mouse models

There are several diseases in which most human symptoms can be mimicked in mouse models including behavioral or cognitive dysfunctions. These diseases are highly associated with neuropathological changes, rather than vascular ones. In addition, aging might be a risk factor of these diseases, but the onset age is relatively younger compared to the diseases introduced in section 2 (described in detail in section 4).

### 4.1 Parkinson’s disease

Parkinson’s disease (PD) is a neurodegenerative disorder characterized by the accumulation of α-Synuclein in the neurons of the substantia nigra and striatum, and damage to dopaminergic neurons (Kalia and Lang, 2015; Balestrino and Schapira, 2020). PD primarily manifests as motor dysfunction, and approximately 40% of PD patients suffer from dementia (Cummings, 1988). The exact cause of dopaminergic neuron impairment in PD remains unclear, highlighting the importance of animal model research in elucidating these mechanisms. Genetic mouse models replicating PD often utilize genes considered to be causative, such as *LRRK2*, *PRKN*, and *PINK1*. Transgenic mouse models of these genes consistently exhibit motor dysfunction, and most of these models show age-related cognitive impairments (Magen and Chesselet, 2011; Magen et al., 2012; Pischedda et al., 2021; Dovonou et al., 2023). Several knock-in mouse models harboring mutations of pathogenic *LRRK2* variants have also been developed. These models typically replicate the characteristic neuronal damage and motor dysfunctions observed in PD (Chang et al., 2022; Dovonou et al., 2023). It is reported that LRRK2 G2019S knock-in mice successfully mimic the neuronal pathology in striatum and cognitive impairments, further contributing to our understanding of the broader impact of PD on cognitive functions (Hussein et al., 2022).

### 4.2 Huntington’s disease

Huntington’s disease (HD) is a disorder resulting from abnormal amplification of CAG repeats in the *Htt* gene, leading to the formation of insoluble aggregates and subsequent neuronal loss (Walker, 2007; Ross and Tabrizi, 2011). HD is characterized by involuntary, dance-like movements of the limbs, known as chorea, cognitive impairments, and psychiatric symptoms (Walker, 2007). Studies using transgenic mice that overexpress *Htt* with amplified CAG repeats have demonstrated the manifestation of motor and cognitive impairments (Lione et al., 1999; Lüesse et al., 2001; Giralt et al., 2011; Kaye et al., 2021). These models have been instrumental in mirroring the symptomatology of HD, providing valuable insights into the disease mechanisms and progression. Similar to transgenic models, knock-in mice carrying HD-like mutations in the *Htt* gene consistently exhibit stable motor and cognitive impairments (Simmons et al., 2009; Giralt et al., 2012; Menalled et al., 2012; Yhnell et al., 2016). This suggests that, similar to PD, HD is a disorder where phenotypic traits are relatively easier to replicate in mouse models.

## 5 The cause that mouse models cannot replicate some diseases and the potential for zebrafish to be model of such diseases

As mentioned above, some diseases can be accurately replicated in mouse models, while others cannot.

A common trait among diseases less effectively modeled in mice is vascular impairment. CADASIL, CARASIL, Fabry disease and RVCL are known as CSVDs, and 80% of AD patients also present with cerebral amyloid angiopathy, a type of CSVD characterized by the accumulation of Aβ deposits in brain arteries (Boyle et al., 2015; Mestre et al., 2017; Greenberg et al., 2020). The pathology of CSVDs is closely related to the glymphatic system, and mice have a more efficient glymphatic system compared to humans, which may contribute to their reduced capacity to accurately phenocopy CSVDs. For diseases like PD and HD, studies showed that the accumulation of abnormal protein such as α-Synuclein and Huntingtin may be involved in cerebrovascular pathology (Drouin-Ouellet et al., 2015; Paul and Elabi, 2022). However, the primary pathology of these diseases is neuronal, a fact supported by the predominant expression of α-Synuclein and Huntingtin in neurons (Young, 2003; Gil and Rego, 2008; Stefanis, 2012; Wong and Krainc, 2017). These suggest that the abnormal aggregated proteins might have a more significant impact on neurons in PD and HD than in CSVDs and AD.

Another commonality is the variability in disease onset. Some previous studies suggested the typical onset age of familial AD ranges from 30s to over 70 years (Percy et al., 1991; Duara et al., 1993; Lopera et al., 1997; Quiroz et al., 2010). Furthermore, while CADASIL has a relatively young onset age, the range is quite broad, with migraines manifesting between 5 and 61 years and lacunar infarcts occurring between 26 and 81 years, indicating that onset at an older age is not uncommon (Tan and Markus, 2016). The wide range of the onset age extending into later years implies that there are individuals who may not exhibit symptoms until they are into old age. On the other hand, the onset age for HD is correlated with the number of CAG repeat amplifications; with over 50 repeats, the onset age is around 20 years (Brinkman et al., 1997; Wexler, 2004). Genetic models of HD in mice possess at least 50 CAG repeats, with some models exhibiting upwards of 150 CAG repeats (Kaye et al., 2021). This suggests that the HD mouse models might exhibit the age-related symptoms in human HD at a younger age. In the case of PD, the typical onset age of familial PD ranges from 20s to 50s, suggesting that familial PD predominantly manifests at a relatively younger age (Spira et al., 2001; Shojaee et al., 2009; Lin et al., 2019). Considering these variations and the relatively short lifespan of mice, mice might not be ideal for accurately replicating human age-related symptoms, potentially limiting their effectiveness in disease modeling.

As zebrafish possesses the less effective clearance capacity in the brain and a long lifespan, diseases that are not fully replicable in mouse models might be more successfully modeled in zebrafish. Although there are currently only a few examples of genetic models used to analyze adult disease states in zebrafish, some studies suggest they offer advantages over mice. In zebrafish with a knockout of *PSEN1*, a risk gene for AD, adult fish exhibit anxiety-like behaviors, a contrast to mice with *PSEN1* knockout, which do not show changes in memory capabilities or anxiety-like behaviors (Saura et al., 2004; Sundvik et al., 2013; Soto-Faguás et al., 2021). Another study established that a zebrafish model expressing human *APP* carrying the Swedish mutation under the control of zebrafish *appb* promotor (Pu et al., 2017). This model showed the Aβ deposition and neuron loss in the telencephalon which controls zebrafish memory, subsequently learning ability was impaired (Pu et al., 2017). In contrast, the mouse model carrying *APP* Swedish mutant showed Aβ deposits but did not exhibit neuronal loss and profound impairment in learning ability, even in their old age (King and Arendash, 2002; Walker et al., 2002).

A recent study established a zebrafish model for Fabry disease by knocking out the *gla* gene encoding α-GalA (Elsaid et al., 2022a). This study found that this zebrafish model could replicate the nephropathy phenotype seen in adult stage, a typical pathology of Fabry disease. Another study showed that the changes in gene expression in the *gla* knockout zebrafish is consistent to that in the *gla* knockout human cell line (Consolato et al., 2022; Elsaid et al., 2022b). However, this zebrafish model has not been analyzed for the neuronal pathology.

In contrast, the genetically modified zebrafish models for CADASIL, CARASIL and RVCL have not been established.

The zebrafish models for PD and HD are well established (Kumar et al., 2021; Doyle and Croll, 2022). These models can replicate human pathology like as mouse models. By employing zebrafish, it is possible to conduct analyses that are not feasible with mice such as drug screening and live imaging (Zhan et al., 2024). Therefore, it is meaningful to develop zebrafish models for diseases for which mouse models already have been well established.

Collectively, as discussed above, zebrafish have potential for modeling age-related neurological diseases particularly accompanied by the vascular pathology, and some zebrafish models of AD show the symptoms that cannot be replicated in mouse models. Further studies are needed to establish the genetic model in zebrafish that closely mirrors human patients. It is crucial that we integrate the insights from various models to unravel the pathomechanism of human age-related neurological diseases.

## 6 Conclusion

Exploring the therapeutic target for the age-related neurological disease is one of the most urgent challenges in today’s global aging society. To address this challenge, researchers should integrate insights obtained from various animal models because each has advantages and disadvantages. We discussed the normal age-related phenotypes of zebrafish which shows similarities to humans, but not mice in aspects such as a lifespan and clearance systems in the brain. In addition, we provided an overview of age-related neurological diseases that can be mimicked in mouse models and those that cannot, using specific examples. Based on the discussion above, it is suggested that diseases that cannot be effectively replicated in mouse models often involve brain vascular pathology. This might be due to the more efficient clearance system in the mouse brain compared to humans and zebrafish. Another reason why zebrafish mimics human age-related neurological disease is their longer lifespan compared to mice. The longevity of zebrafish enables replication of symptoms and pathologies that worsen with age. In conclusion, zebrafish has a great potential for mimicking human age-related neurological disease, due to their similar clearance system in the brain and lifespan to humans.

## Author contributions

TK: Conceptualization, Resources, Writing – original draft, Writing – review and editing. TM: Writing – review and editing. AT: Writing – review and editing. MI: Conceptualization, Resources, Supervision, Writing – review and editing.
